# Validation of Cadherin HAV6 Peptide in the Transient Modulation of the Blood-Brain Barrier for the Treatment of Brain Tumors

**DOI:** 10.3390/pharmaceutics11090481

**Published:** 2019-09-17

**Authors:** Babu V. Sajesh, Ngoc H. On, Refaat Omar, Samaa Alrushaid, Brian M. Kopec, Wei-Guang Wang, Han-Dong Sun, Ryan Lillico, Ted M. Lakowski, Teruna J. Siahaan, Neal M. Davies, Pema-Tenzin Puno, Magimairajan Issai Vanan, Donald W. Miller

**Affiliations:** 1 Research Institute in Oncology and Hematology, University of Manitoba, Winnipeg, MB R3E 0V9, Canada; 2 Department of Pharmacology and Therapeutics, University of Manitoba, Winnipeg, MB R3E 0T6, Canada; 3 College of Pharmacy Pharmaceutical Analysis Laboratory, University of Manitoba, Winnipeg, MB R3E 0V9, Canada; 4 Department of Pharmaceutical Chemistry, Faculty of Pharmacy, Kuwait University, Safat 13110, Kuwait; 5 Department of Pharmaceutical Chemistry, University of Kansas, Kansas, KS 66205, USA; 6 Kunming Institute of Botany, Kunming 650201, Yunnan, China; 7 Pharmacy and Pharmaceutical Sciences, University of Alberta, Alberta, AB T6G 2R3, Canada; 8 Department of Pediatrics and Child Health, University of Manitoba, Winnipeg, MB R3T 2N2, Canada

**Keywords:** blood-brain barrier (BBB), drug delivery, transient modulation, HAV6 cadherin peptide, adenanthin, magnetic resonance imaging (MRI), medulloblastoma

## Abstract

The blood-brain barrier (BBB) poses a major obstacle by preventing potential therapeutic agents from reaching their intended brain targets at sufficient concentrations. While transient disruption of the BBB has been used to enhance chemotherapeutic efficacy in treating brain tumors, limitations in terms of magnitude and duration of BBB disruption exist. In the present study, the preliminary safety and efficacy profile of HAV6, a peptide that binds to the external domains of cadherin, to transiently open the BBB and improve the delivery of a therapeutic agent, was evaluated in a murine brain tumor model. Transient opening of the BBB in response to HAV6 peptide administration was quantitatively characterized using both a gadolinium magnetic resonance imaging (MRI) contrast agent and adenanthin (Ade), the intended therapeutic agent. The effects of HAV6 peptide on BBB integrity and the efficacy of concurrent administration of HAV6 peptide and the small molecule inhibitor, Ade, in the growth and progression of an orthotopic medulloblastoma mouse model using human D425 tumor cells was examined. Systemic administration of HAV6 peptide caused transient, reversible disruption of BBB in mice. Increases in BBB permeability produced by HAV6 were rapid in onset and observed in all regions of the brain examined. Concurrent administration of HAV6 peptide with Ade, a BBB impermeable inhibitor of Peroxiredoxin-1, caused reduced tumor growth and increased survival in mice bearing medulloblastoma. The rapid onset and transient nature of the BBB modulation produced with the HAV6 peptide along with its uniform disruption and biocompatibility is well-suited for CNS drug delivery applications, especially in the treatment of brain tumors.

## 1. Introduction

Chemotherapy and more recently targeted therapy directed at specific tumor targets, are important modalities for treatment of pediatric brain tumors. Numerous chemotherapeutic drugs and small molecule inhibitors that demonstrated significant antitumor activity in preclinical studies have failed in human clinical trials [1]. These disappointing results can partially be explained by the ineffective drug delivery to the CNS [1]. The anatomical features posed by the various specialized physiological barriers (blood-brain barrier-BBB, and blood tumor barrier) together with the various active efflux transporters expressed contribute to drug failure because of the inability to reach the desired target at a therapeutically relevant concentrations [1]. The BBB prevents many drug molecules within the systemic circulation from entering the brain. Even small hydrophobic drugs that could otherwise partition into the plasma membranes and diffuse intracellularly through the vasculature have limited brain penetration due to the presence of multiple efflux transporters [2,3] and drug metabolizing enzymes [4,5] within the brain’s endothelial cells. For hydrophilic drugs and macromolecules, the complex tight junctions that form between the brain endothelial cells limits the paracellular diffusion pathway for brain penetration [6]. Indeed, it has been suggested that molecules with hydrodynamic diameters larger than 11 Å or molecular weight of 500 D are too large to pass through the BBB [6,7]. Under pathological conditions, such as brain tumors, the BBB within the tumor area can become leaky, resulting in contrast enhancement on magnetic resonance imaging (MRI) of the tumor [8]. Indeed, the leakage of the MRI contrast media into the tumor site has been cited as evidence for a leaky BBB in brain tumors [9,10]. However, studies in mice have shown that despite the altered BBB integrity within the tumor site at later stages of tumor development, the BBB is still functional and limits solute and drug permeability in and around the tumor [11]. Furthermore, patients with primary brain tumors have clinically significant regions of tumor with an intact BBB and failure to deliver effective therapy to all regions of glioblastoma tumor contributes to treatment failure [12].

The cadherin proteins found within the adherens junction have an important role in the BBB for establishing cell–cell contact and contributing to the tight junction complex. The formation of homodimer complexes of cadherin proteins on adjacent brain microvessel endothelial cells act to physically restrict the paracellular passage of solutes from the blood to the brain extracellular environment [6,7]. Molecularly, the extracellular (EC) domain consists of five tandem repeated units (EC-1 to EC-5). Within the EC1 domain, the highly conserved region of His-Ala-Val (HAV) is crucial for the formation of the cis-dimer formation of cadherins. Synthetic peptides targeting this HAV region sequence of the EC1 domain display concentration-dependent binding to E-cadherin molecules and can prevent homodimer complex formation in brain microvessel endothelial cells [13]. We have previously [14] shown that systemic (intravenous, IV) administration of synthetic E-Cadherin peptide in Balb/c mice caused a reversible disruption of BBB integrity and enhanced the accumulation of permeability markers of various molecular weights ((low molecular weight gadolinium diethylene-triamine-penta-acetate (Gd-DTPA), larger molecular weight infrared Dye (IRDye800CW) and a drug efflux agent, rhodamine 800 (R800)). The magnitude of increase in BBB permeability observed ranged from two-fold to five-fold (depending on size and chemical properties of imaging agent), was rapid, (occurred within 3−6 min following injection of the peptide) and reversible, with complete barrier integrity being restored within 60 to 90 min of injection [14]. Additional studies with both linear and cyclized cadherin peptides demonstrated that the duration and magnitude of BBB opening could be selected based on the stability and binding affinity of the cadherin peptides [14,15,16]. These studies provided the impetus for use of cadherin peptides to improve brain delivery of therapeutics.

Medulloblastoma (MBL) is the most common malignant brain tumor of childhood and accounts for 10% of all deaths from childhood cancer [17]. Current management consists of surgical resection followed by ionizing radiation (IR) and chemotherapy. Outcome for high-risk patients remains relatively poor, with a five-year event-free-survival of 25–40%. MBL comprises at least four distinct molecular subgroups, with Group-3 having by far the worst prognosis with a 5-year survival of approximately 30% [17]. To date, no specific targeted therapy is available for Group-3 MBLs. Peroxiredoxin-1 (PRDX1) is a multifunctional protein that catalyzes hydrogen peroxide into water and oxygen and thereby prevents free radical mediated oxidative stress [18]. Besides mediating radiation resistance in several cancers, PRDX1 has been suggested to play a role in chemotherapy resistance, cell differentiation, proliferation and apoptosis [18]. We recently validated PRDX1 as a therapeutic target in Group-3 MBL [19]. Ade, a diterpenoid compound isolated from the leaves of *Isodon adenantha* inhibits the peroxidase activity of PRDX 1 and 2 [20]. Adenanthin has shown therapeutic activity in several cancers, like leukemia and liver cancer [20,21], but there is an absence of data pertaining to its effectiveness in treating brain tumors. In the present study, we demonstrated that Ade, when administered alone, did not cross the BBB and was ineffective in treating D425 tumors in a mouse orthostatic medulloblastoma tumor model. However, concurrent use of HAV6 peptide to transiently open the BBB resulted in Ade entry into the brain and significantly prolonged the survival of mice bearing Group-3 MBL tumors. Those tumor bearing mice receiving HAV6 showed no additional adverse responses to the treatment compared to mice receiving placebo. These findings provide proof-of-principle for the use of cadherin peptides in the modulation of BBB permeability and improved treatment of brain tumors.

## 2. Materials and Methods

### 2.1. Chemicals and Reagents

Gadolinium diethylene-triamine-penta-acetate (Gd-DTPA) was obtained from Berlex (Lachine, QC, Canada) and used as a contrast agent for MRI monitoring of BBB permeability. Analytical grade Formic acid and HPLC Grade Acetonitrile were purchased from Fisher Scientific. Ultrapure water from a Milli-Q^®^ system (Millipore, Billerica, MA, USA) was used for mobile phase. All other reagents and chemicals were purchased from Sigma Chemical Company (St. Louis, MO, USA).

### 2.2. HAV6 Peptide Synthesis

The HAV6 peptide (Ac-SHAVSS-NH_2_) was synthesized using solid phase Fmoc-chemistry in a Tribute peptide synthesizer (Gyros Protein Technologies, Tucson, AZ), as described previously [13]. After removal from the resin, the peptide was purified using a semi-preparative C18 column in HPLC. The pure fractions were pooled and lyophilized. The purity of the peptide was higher than 98%, as determined by C18 analytical HPLC. The identity of the peptide was confirmed by mass spectrometry.

### 2.3. Adenanthin Source and Formulation

Adenanthin was isolated from the dried aerial parts of the leaves of Isodon Adenanthus Hara, as described previously [20]. Ade (MW 490.549 g/mol) was reconstituted in 1% DMSO in phosphate buffered saline (PBS) (KCl; 2.66 Mol, KH_2_PO_4_; 1.47 Mol, NaCl; 137.93 Mol, Na_2_HPO_4_-7H_2_O; 8.06 Mol; pH 7.40) at a final concentration of 1 mg/µL to obtain a 10X stock. The solution was diluted to 0.1 mg/µL in physiological saline and was administered to animals so that each animal got a final concentration of 10 mg/kg body weight.

### 2.4. Animals and Ethics Statement

All experiments described in this study were done at the University of Manitoba and the Research Institute in Oncology and Hematology, as described in animal use protocol 13-051 approved by the Central animal care committee at the University of Manitoba in accordance with the guidelines provided by Canadian council on Animal care on 18 November 2015. All surgical procedures were performed under anesthesia induced and maintained by Isoflurane and every effort to minimize pain, suffering and a reduction in the numbers of animals used were made.

### 2.5. MRI Imaging of BBB Permeability with HAV6 Peptide

The HAV6 peptide-induced BBB permeability enhancement in Ncr (nu-/nu-) mice was assessed using MRI and Gd-DTPA contrast agent as described previously [11,14]. Briefly, the mice were anesthetized and placed in a 7 Tesla small animal Bruker Biospect MR with a 21 cm bore and 2.5 × 2.5 cm^2^ field of view for spectroscopy. Once secured into MRI, a series of T1-weighted coronal images of the mouse brain were obtained before Gd-DTPA delivery as a contrast agent to acquire background images of the mouse brain. Mice were then administered Gd-DTPA contrast agent (0.4 mmol/kg) along with either HAV6 cadherin peptide (0.01 mmol/kg) or vehicle (PBS) via bolus tail vein injection, and T1-weighted coronal images of the entire brain were obtained at 3 min intervals throughout a 21 min imaging session. After the first imaging session, a second dose of Gd-DTPA was administered and T1-weighted images obtained for an additional 30 min. Quantitative assessment of Gd-DTPA enhancement in various regions of interest in the brain were obtained using Marevisi 7.2 software (Institute for Biodiagnostics, National Research Council, Canada). Changes in Gd-DTPA intensity in the brain as a function of time and treatment were determined using a percent difference analysis of brain slice images obtained as described previously [11,14,22] using the following formula.

(post-Gd-DTPA-T1-weighted image − pre-Gd-DTPA-T1-weighted image) ÷ pre-Gd-DTPA-T1-weighted image) × 100.

### 2.6. LC-MS/MS Analysis and Conditions

The impact of HAV6 on the BBB permeability of Ade was assessed using the brain to plasma ratio of Ade. For these studies, healthy female Ncr (nu–/nu–) mice were randomly selected to receive bolus injections (1 mL/kg) of either vehicle (Physiological saline), Ade alone (10 mg/kg) or a combination of HAV6 (0.010 mmol/kg) with various concentrations of Ade (5, 10 and 15 mg/kg,) via tail vein injections. The mice were then sacrificed 20 min after the injections, and blood and various tissues, including the brain, were subjected to LC-MS/MS analysis.

#### 2.6.1. Sample Preparation

The extractions of Ade from the plasma and brain tissues were done using acetonitrile and a mixture of acetonitrile with deionized water at a ratio of 2:1 respectively. Frozen plasma samples were thawed followed by the addition of acetonitrile at a ratio of 2:1. For the brain tissue, the samples were weighed, thawed and homogenized with the addition of acetonitrile and water (2:1) at the volume of 5× the weight of the samples. The samples were then vortex mixed continuously for 5 min at a temperature of 4 °C, followed by centrifugation at 4 °C for 10 min at a speed of 10,000 g (for brain) and 1875 g (for plasma). The supernatants were collected and evaporated using a flow of nitrogen gas. Adenanthin in methanol was added to 200 μL blank plasma or blank brain homogenate to achieve concentrations of 0.1, 1, 10 and 100 μg/mL for standardization. Samples and standards were treated with 400 μL cold acetonitrile (−20 °C) to precipitate proteins and were vortexed for 2 min, and centrifuged at 15,000 rpm for 5 min. The supernatants were transferred to new tubes and evaporated to dryness using a Savant SPD1010 SpeedVac Concentrator (Thermo Fisher Scientific, Inc., Asheville, NC, USA) without heat. The dried samples were reconstituted in 400 μL of 50% aqueous methanol with 0.1% formic acid and centrifuged at 15,000 rpm for 5 min. The supernatant was transferred to HPLC vials and 10 mL injected into the LC-MS/MS system.

#### 2.6.2. LC-MS/MS Analysis

A Shimadzu Nexera ultra high performance liquid chromatography system connected to an 8040 triple-quadrupole mass spectrometer (LC-MS/MS) (Shimadzu, Kyoto, Japan) was used to measure Ade. A dual Electrospray ionization (ESI) / atmospheric pressure chemical ionization (APCI) ion source was used in the positive mode multiple reaction monitoring (MRM) for the sodium adduct [M^+^Na^+^] of Ade with the transition 513.2 > 453.1 *m*/*z* using a collision energy of 30 eV. The desolvation line temperature was 250 °C; the heating block temperature was 400 °C; the nebulizing gas flow was 2 L/min; and drying gas flowed at 15 L/min. An XTerra^®^ MS C18 (3.5 μm, 2.1 × 50 mm) (Waters Corporation, Milford, MA, USA) column was used with 0.3 mL/min flow rate at 30 °C. The mobile phases A (50% aqueous methanol with 0.1% formic acid) and B (85% aqueous methanol), and 0.1% formic acid were used in a step gradient starting at 100% A for 1 min, then stepping to 100% B for 3 min and stepping back to 100% A for 2 min before the next injection.

### 2.7. MBL Xenograft and Animal Experiments

#### 2.7.1. Cell culture and authentication

Early passage Group-3 Medulloblastoma cells (D425-MED) were a kind gift from Dr. Darell Bigner, Duke University, and have been described previously [23]. Cells were cultured at 37 °C in Minimum Essential Medium (Richter’s modification; ThermoFisher/Gibco™ Markham, ON, Canada) supplemented with fetal bovine serum (10%); ThermoFisher/Gibco™) in a humidified incubator with 5% CO_2_. These cells were transduced by a lentiviral infection to stably express firefly luciferase. The resulting D425-Med-Luc cells were authenticated and validated by morphology and phenotype during recovery from the frozen stock and growth characteristics during culture.

#### 2.7.2. Animal Experiments

An orthotopic Group-3 medulloblastoma mouse model was used to evaluate Ade effectiveness, both alone and in combination with HAV6 peptide for transient BBB disruption. Outbred homozygous nude (NCr nu; Foxn1nu/Foxn1nu, female mice < 4 weeks old) mice with jugular vein access (C20PU-MJV1301; Instech Laboratories, Inc.) and vascular access button (VAB62BS/25; Instech Laboratories, Inc) were obtained from Charles River Laboratories, Saint Constant, QC, Canada. Group 3 MBL (D425-Med-Luc) cells were implanted into the cerebella of these mice as described previously [24]. All mice were serially imaged on an IVIS^®^ spectrum (Perkin Elmer, Waltham, MA), small animal imager, starting on day-7 post tumor cell implantation to confirm the presence and extent of tumor growth using bioluminescence as described previously [25]. Tumor bearing mice were randomly assigned into one of the following treatment groups (*n* = 6): (1) placebo (physiological saline); (2) Ade (10 mg/kg) alone; or (3) HAV6 cadherin peptide (0.01 mmol/kg) with Ade (10 mg/kg). All treatments were given as intravenous injection via the jugular access catheter. Treatments were begun on day-10 post tumor cell implantation. Each treatment cycle consisted of 3 consecutive days of treatment followed by one day of rest, with a maximum of 5 cycles given to each mouse. Mice were assessed daily for tumor progression using humane endpoints, including weight loss (>25% from original weight on day of tumor cell implantation), limb paralysis, locomotion and seizure activity. Tumor progression was monitored by bioluminescence imaging every 3 days starting at day 7-post tumor cell implantation as described in Baumann et al. [25]. Upon reaching humane endpoints tumor-bearing mice were euthanized by cardiac perfusion under iso-flurane anesthesia and the brains were removed and used for histology. Survival data was recorded and Kaplan-Meier curves were generated using Prism6 (V6.0h; GraphPad Software, Inc.; San Diego, CA, USA).

### 2.8. Immunohistochemistry

When mice bearing tumors presented with moribund phenotype, they were euthanized as described above. Subsequently, brains from each animal were extracted and fixed in 10% phosphate buffered formalin (pH 7.4) for 24 h. They were dehydrated by immersion in graded alcohol solutions for 10 min each (50%, 70%, 80% NS 95%) and for 3 changes in 100% ethanol. Tissue was embedded in paraffin and serial sections (6 μm) of the brain were collected on positively charged glass slides. Paraffin was melted and removed from slides by placing in an incubator at 60 °C for 30 min. Subsequently, slides were immersed in Xylene for 3 min, twice and rehydrated by immersion in a series of graded alcohol solutions for 3 min each (100%, 95%, 80%, 70%, 50%) and were finally washed in deionized water. Immediately, slides were immersed in 1X universal antigen retrieval reagent (ab208572, abcam, Cambridge, MA, USA) and incubated for 20 min at 95 °C in a programmable pressure chamber (Decloaking chamber NxGen, Biocare medical) following the manufacturer’s instructions. Slides were stained to detect the expression of PRDX1 (abcam; Anti-PRDX1 antibody ab41906), γ-H2A.X (abcam; Anti-γ-H2A.X phosphorylated S139 antibody; ab2893), Ki 67 (abcam; Anti Ki67 antibody; ab 15580), 53BP1 (abcam; Anti-53BP1 antibody; ab21083) and cleaved caspase 3 (abcam; Anti-cleaved caspase 3 antibody; ab13847) using an HRP/DAB detection method by using a mouse and rabbit specific HRP/DAB detection IHC kit (abcam; ab80436-EXPOSE mouse and rabbit specific HRP/DAB detection IHC kit), following manufacturers recommendations with minor modifications. Using normal human cerebellar cortex tissue as control, the optimal concentrations of each antibody and the incubation time with DAB substrate was empirically determined to be 1:1000 with an overnight incubation at 4 °C. Subsequently, stained tissue was imaged on a Cytation 5, a cell imaging multi-mode reader (Biotek Instruments Inc, Winooski, VT, USA), and color bright field images were captured on a 16-bit Sony charge coupled device Camera and Gen5 V.3.04 software. An image montage was obtained using a 2.5X objective Meiji, Plan Achromat, working distance (WD) of 6.2 and numerical aperture (NA) of 0.07 to image the entire section. A region of interest was manually imaged using a 20X objective, Plan Flourite, WD 6.6 and NA 0.45. Color images were processed using Gen5 V.3.04. Color images were saved as high-resolution JPEG images and were imported into Adobe Photoshop Creative Suite CS5, Adobe, San Jose, CA, USA to form image panels and additional illustration.

### 2.9. Cytotoxicity Studies

The D425-Med-luc cells were seeded onto a 96-well plate at a density of 5000 cells per well. Twenty-four hours after seeding, HAV6 (final concentration of 0.01 mM) or phosphate buffered saline (10 μL) was added to the culture media and cells were cultured for 48 h. Viable cells remaining at the end of the experiment were fixed with 4% paraformaldehyde, nuclei were stained with Hoechst 33342 (H3570; Fisher Scientific Inc., St. Loius USA) and imaged on a Cytation 5, a cell imaging multi-mode reader (Biotek Instruments Inc., Winooski, VT, USA) assay as previously described [26].

### 2.10. Statistical Analyses

Raw data was imported into Prism 6 GraphPad and analysis of variance was performed with post hoc comparison of the means as indicated in figure legends. Survival curve analysis and analysis of variance were performed with post-hoc nonparametric log-rank (Mantel-Cox) tests for significance. Values were represented as mean ± SEM unless otherwise indicated in the figure legends.

## 3. Results

### 3.1. HAV6 Peptide Transiently Increases BBB Permeability In-Vivo

**Figure 1:**
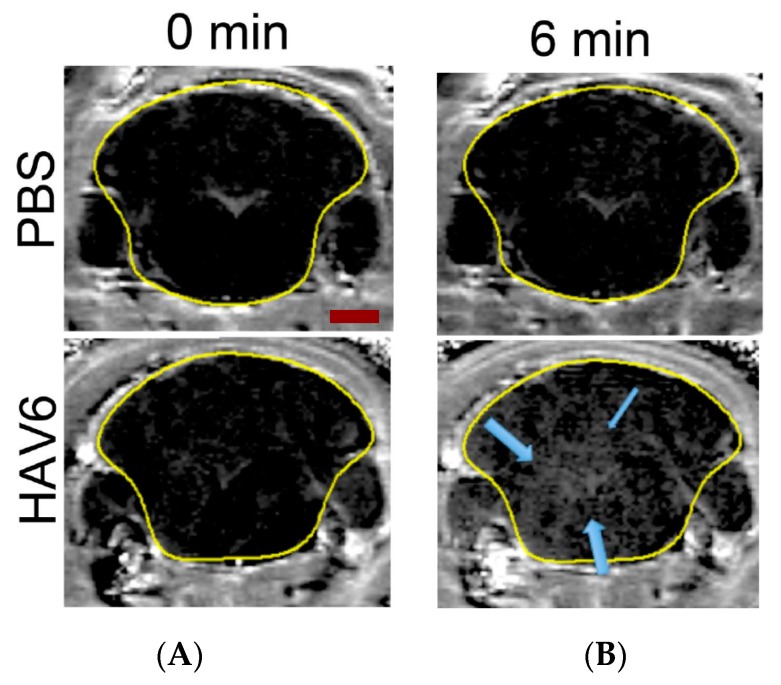
Representative MRI T1-weighted images of mouse brain taken at 0 min (**A**) and 6 min (**B**) following the injection of Gd-DTPA in control (PBS) and HAV6 (0.01 mmol/kg) treated mice. Blue arrows indicate regions of Gd-DTPA enhancement following the injection of the peptide. Scale bar represent 2 mm.

In order to test if the novel HAV6 peptide would increase the BBB permeability, we injected mice with HAV6 peptide or vehicle and imaged their brains using MRI and Gd-DTPA contrast. As seen in Figure 1, the BBB integrity was not compromised in vehicle treated mice as shown by similar Gd-DTPA contrast enhancement in representative brain slice images at time 0 (prior to the injection of vehicle) and at 6 min following vehicle injection (Figure 1A,B, respectively). These coronal slice images show minimal Gd-DTPA, within the brain, consistent with its low BBB penetrance under normal conditions (Figure 1A,B). However, there was a significant increase in the signal intensity of the Gd-DTPA contrast agent in the brain (represented by white-gray appearance indicated by blue arrows) following IV injection of HAV6 peptide (0.01 mmol/kg) (Figure 1).

**Figure 2:**
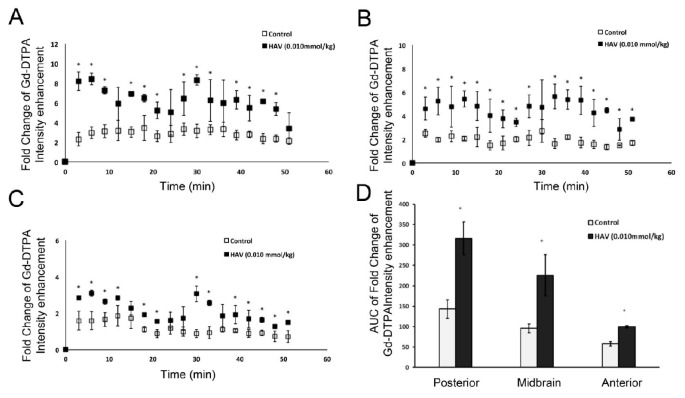
Analysis of pixel intensity for Gd-DTPA from T1-weighted images normalized to the pixel intensity at time 0 of the injection in (**A**) the posterior, (**B**) midbrain and (**C**) anterior regions of the brain. (**D**) Area under the curve for Gd-DTPA obtained from T1-weighted images over the span of 51 min. * *p* value < 0.05 in comparison to control group. Values represent the mean ± SEM for four mice per treatment group.

Indeed, quantitative assessment of Gd-DTPA contrast enhancement across the various brain regions indicated an approximately 2–4-fold increase in Gd-DTPA intensity in the HAV6 treatment group compared to control, and this increase was most apparent at 6–9 min following the injection of the HAV6 peptide (Figure 2A–C) and was consistent with our previous findings [14]. Furthermore, while the signal intensity for Gd-DTPA varied as a function of brain region examined, the increases in Gd-contrast enhancement in response to HAV peptide was observed in all regions of the brain examined, as indicated by examining the resulting area under the curve (AUC) from the Gd-DTPA intensity versus time plots taken over a 51 min period following HAV6 administration (Figure 2D). These studies confirmed the rapid onset and relatively brief BBB modulation following the administration of HAV6 cadherin peptide.

### 3.2. In Silico and In Vivo Determinations of Ade Permeability in the BBB

**Figure 3:**
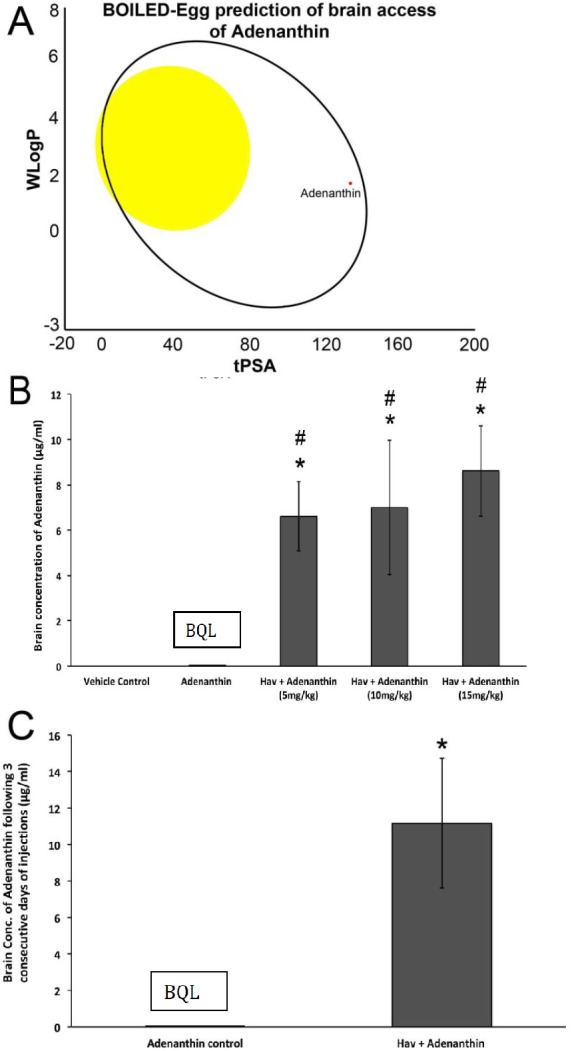
(**A**) Visual representation of in silico prediction of brain access and availability of Ade using the BOILED-Egg model, adapted from Daina, A.; Zoete, V. A BOILED-Egg to predict gastrointestinal absorption and brain penetration of small molecules. *ChemMedChem* **2016**, *11*, 1117–1121. [27]. The tPSA (*x*-axis) and WLogP (*y*-axis) values are plotted and the intersect of those values can predict brain access if it falls within the “yellow yolk”. If the intersect lies within the white ellipse, that indicates its gastrointestinal availability. Based on this model, Ade may be available via a gastrointestinal method of delivery but may not access the brain. Quantitative analysis of Ade in the brain using LCMS under control conditions and following treatment with HAV6 after (**B**) a single treatment of different Ade doses and (**C**) three consecutive daily treatments. * *p*-value < 0.05 in comparison to control group. # *p*-value < 0.05 compared to Ade group only. Values represent the mean ± SEM for four mice per treatment group.

We employed the BOILED-Egg model (the Brain Or IntestinaL EstimateD permeation predictive model) [27] to predict if Ade would cross the BBB and be available in the brain. The lipophilicity (determined by the n-octanol/water partition coefficient-WLogP) and polarity (determined by the topological polar surface area-tPSA) values for Ade were calculated using the free online chemical property calculation service provided by www.molinspiration.com. The canonical SMILES (Simplified Molecular-Input Line-Entry System) and molecular structure of Ade was determined from a PubChem search (https://pubchem.ncbi.nlm.nih.gov/compound/Ade). Applying this model, we show that Ade has a low probability of crossing the BBB (Figure 3A) [27].

**Figure 4:**
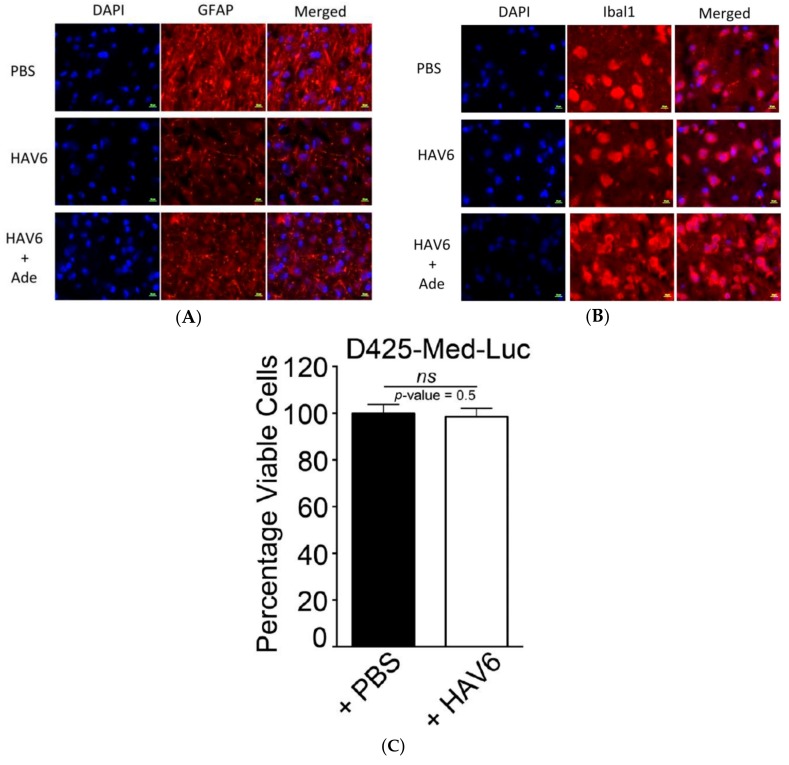
Representative immunofluorescence images of healthy mouse brain to detect (**A**) GFAP and (**B**) Ibal1 following the three consecutive daily treatments of PBS, HAV6 and the combination of HAV6 + Ade. The presence of HAV6 has no impact on the expression level of GFAP and Ibal1 compared to PBS control. Scale bar in bottom represent 10 µm. (**C**) Graphical representation of viability of D425-Med-Luc cells treated with either vehicle control (black bars; PBS) or HAV6 peptide (0.01 mmol; white bars). Values represent mean ± SEM of viable cells from eight monolayers per treatment group. Data are expressed as the percent viable cells compared to D425-Med-Luc cells receiving culture media alone. HAV6 has no negative impact on cell viability; *t*-test indicated *p*-value is 0.496952 and is non-significant (*ns*).

Consistent with the initial in silico assessment, in vivo analysis in mice revealed that Ade, when administered alone, had limited BBB permeability, with brain concentrations below the quantitative limit (BQL; 0.1 mg/mL) (Figure 3B). However, when Ade was co-administered with HAV6-cadherin peptide, there was a significant and substantial increase in brain concentrations of Ade (Figure 3B). The resulting Ade brain concentrations were statistically similar at all doses of Ade examined (Figure 3B), reaching levels of approximately 10–16 μM. In order to determine the tolerability of a complete cycle of therapy, mice were administered HAV6 cadherin peptide on 3 consecutive days. After three consecutive days of treatment Ade levels in the brain were detectable but below the quantitative limit of analysis (Figure 3C). However when Ade was combined with the HAV6 peptide, brain concentrations of Ade were approximately 22 μM (Figure 3C). Furthermore, analysis of brain samples from mice treated with consecutive injections of either HAV6 or combination of HAV6 and Ade showed no evidence of toxicity (Figure 4A,B), as indicated by absence of astrogliosis and neuro-inflammation (microglia activation) following a complete cycle of HAV6 treatment to enhance BBB delivery of Ade.

### 3.3. HAV6 Cadherin Peptide Improves Tumor Response to Ade

**Figure 5:**
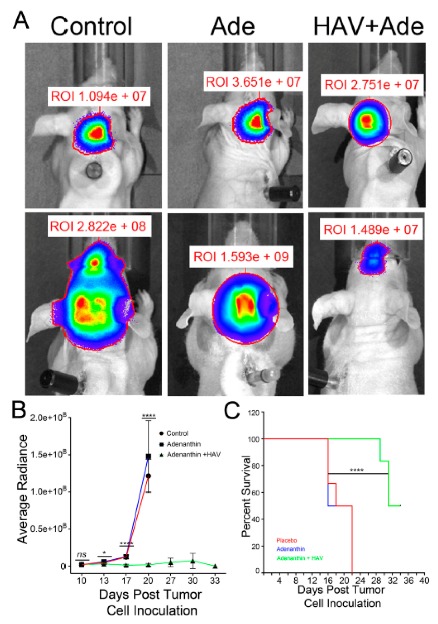
(**A**) Representative bioluminescence image of tumors in mice prior to the treatments (top panels) and following a 5-cycle treatment of control, Ade, or combination of HAV6 and Ade (bottom panel). (**B**) Quantitative analyses of the bioluminescence from tumors as outlined in the regions of interest (ROI) and normalized to background intensity for all tumor mice receiving control, Ade, or combination of HAV6 and Ade. (**C**) Kaplan-Maier survival curve of MBL tumor mice following the five-cycle treatment of control, Ade, or combination of HAV6 and Ade. Each treatment group consisted of six mice.

To functionally demonstrate that the HAV6 peptide can transiently disrupt the BBB and facilitate delivery of Ade to the brain, and therapeutically target PRDX1 we treated mice bearing Group 3 D425-Med-Luc tumors with either Ade alone or Ade in combination with HAV6 peptide. The mice receiving placebo or Ade alone had a median survival of 20 and 19 days post tumor cell injection respectively (Figure 5C). Furthermore, none of the mice in the placebo or Ade treatment group were able to complete the entire five cycles of treatment or survive past 22 days. In contrast, mice receiving HAV6 peptide and Ade showed significant improvement in tumor response (Figure 5A,B) compared to placebo or Ade alone, with median survival of 30 days post-tumor cell injection (Figure 5C). In addition, half of the mice receiving HAV6 peptide and Ade completed all five cycles of treatment and survived to 45 days post tumor cell injection with no sign of a tumor, as indicated by bioluminescence imaging.

**Figure 6:**
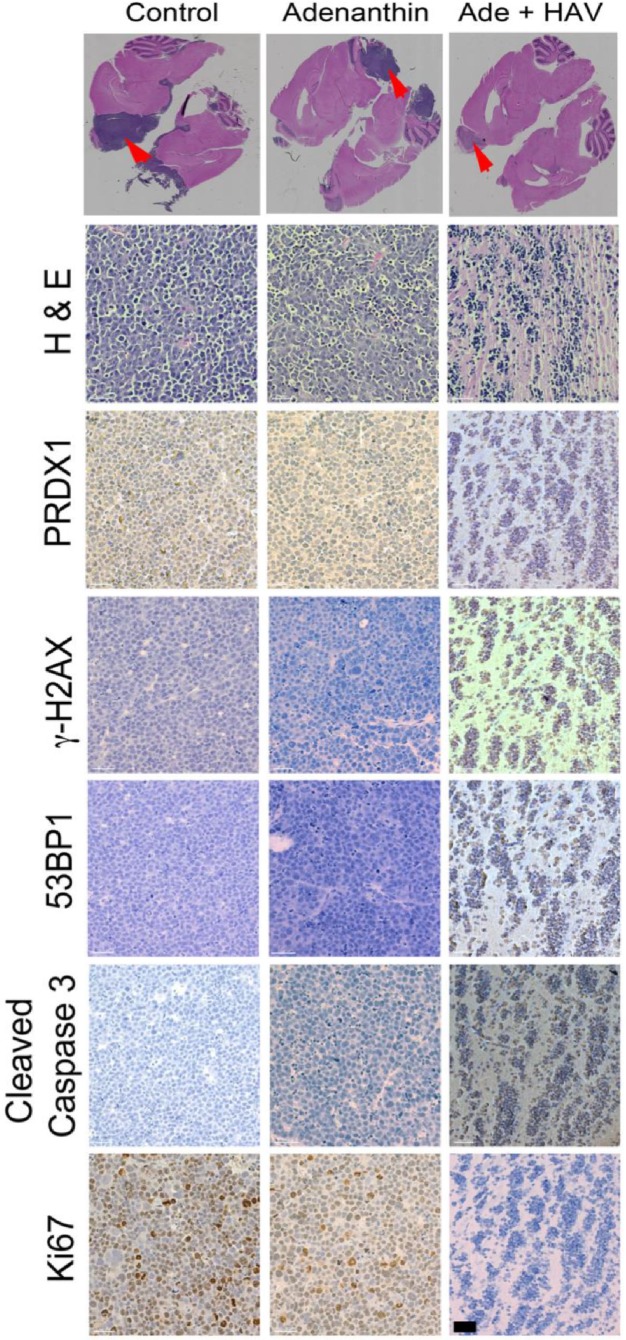
Magnification scans of whole brain sections stained with Hematoxylin and Eosin (H&E) of representative animals that received placebo (control), Ade or a combination of Ade and HAV peptide (Top panel). Arrows indicate those regions that were further examined for various tumor markers (Bottom Panel). White line indicates a scale bar of 100 μm.

At the cellular level, tumor cells were positive for PRDX1, an ROS scavenging enzyme that is the molecular target of Ade (Figure 6). Mice receiving placebo or Ade alone showed increased cellular proliferation, compared to the animals that received both HAV6 peptide and Ade (Figure 6). Consistent with the increased tumor responsiveness observed in mice treated with Ade and HAV6 peptide, there was increased intensity of staining observed for γ-H2A.X and 53BP1, surrogate markers of DNA double strand breaks in the tumor cells (Figure 6). Similarly there was increased apoptotic death in tumor cells that received Ade and HAV6. Taken together, the data suggests that HAV6 peptide facilitates the entry of Ade into the brain, allowing Ade to reach therapeutically significant levels that can affect tumor cell death.

## 4. Discussion

The development of drugs for the treatment of many CNS disorders has long been a difficult process, in part due to the inability of the drugs to reach relevant therapeutic concentration in the brain and the spinal cord. This limited access to the brain and spinal cord for many drugs is a direct result of the protective properties of the BBB and blood cerebral spinal fluid barrier (BCSFB). These barriers are composed of epithelial (BCSFB) or endothelial (BBB) cells with tight junctions and active efflux transporters that together restrict both the paracellular and transcellular passages of solutes into the brain [28,29]. While these barriers protect the brain and spinal cord from exposure to potential neurotoxic agents, under pathological conditions, such as a brain tumor, these barriers represent significant obstacles to therapeutic agents intended for treating these disorders. Several approaches have been used in an effort to circumvent these barriers to improve drug penetration into the brain. For treatment of brain tumors, transient disruption of the tight junctions of the BBB allowing more drug to penetrate have been a successful approach both in preclinical and clinical studies [30,31,32,33]. This can be done through the use of high concentration of osmotic agents (i.e., a high concentration of mannitol) or bradykinin analogues [30,31,32,33], or more recently, through focused, high-intensity ultrasound [34,35,36].

Approaches such as osmotic disruption and focused, high-intensity ultrasound, have shown promise in preclinical studies and have been used in the clinic to enhance chemotherapeutic drug delivery to brain tumors. However, the long duration of BBB disruption (up to 8 h or more) can lead to neurotoxicity and inflammation [37,38]. A long recovery time (6–72 h) has also been reported for focused high intensity ultrasound [36,39]. Other approaches, such as use of bradykinin receptor agonists, result in a shortened BBB disruption timeframe compared to either hyperosmotic or ultrasound disruption, but lack clinical effectiveness, due in part to a non-uniform distribution of bradykinin receptors in the brain microvasculature, resulting in a non-uniform distribution of the drugs [14,40]. Finally, none of the current transient BBB disruption approaches provide much control over the magnitude of BBB opening. Clearly the limitations of the existing transient disruption approaches highlight the need for alternative methods of achieving a controlled BBB disruption profile that is able to uniformly enhance BBB permeability to improve drug delivery to the brain.

Synthetic HAV6 peptide disrupts the BBB by inhibiting homodimer interactions between E-cadherin, an essential protein that forms the adherens junctions of the BBB [13,40]. Based on our previous studies, HAV6 peptide-induced changes in BBB permeability were apparent within 3 min with the HAV6 peptide being able to produce similar magnitudes of BBB disruption in all regions of the brain examined [14,15,16]. Furthermore, these studies also demonstrated that the magnitude of disruption could be modulated with some linear cadherin peptides, such as HAV6, producing BBB openings for small and medium sized permeability markers for short time periods, while other cyclic cadherin peptides had longer durations of action and enhanced BBB penetration of large macromolecules [14,15,16]. Based on these previous findings, the HAV6 cadherin peptide was selected for the present study, as it demonstrated short durations of BBB opening for small molecules without wholesale opening of the BBB to large macromolecules like albumin.

The initial characterization of HAV6 peptide mediated BBB disruption in nude mice was examined using Gd-DTPA and MRI. Following HAV6 peptide administration, there was an approximately two to four-fold enhancement in Gd-DTPA contrast throughout all areas of the brain examined. Both the magnitude and time frame of BBB disruption mediated by HAV6 peptide in the current study were consistent with our previous findings [14]. The ability to produce a rapid and uniform disruption of BBB with the HAV6 peptide is crucial for eliminating the “sink effect” that is often observed with non-uniform disruption approaches that result in reduced drug concentrations at the tumor site due to the diffusion of drugs from an area of high concentration to an area of low concentration. Furthermore, the Gd-DTPA contrast MRI studies also allowed a quantitative assessment of the duration of BBB modulation produced with the HAV6 peptide and indicated that the time frame for BBB opening was of short duration.

The anticancer properties of Ade were originally reported by Liu et al. [19] in studies examining a series of diterpenoids that formed adducts with PRDX1 and PRDX2. While initially reported as a PRDX selective inhibitor, there is evidence of potential inhibitory activity of Ade in both the thioredoxin and protein disulfide isomerase enzymes that are involved in redox metabolism in cells [41]. Studies in multiple cancer cells indicate Ade has cytotoxic responses linked with PRDX inhibition [19,20,21,42]. Ongoing work in our laboratory has shown that PRDX1 is overexpressed in Group-3 MBL and may contribute to radiation resistance and poor outcomes [19]. While the effects of Ade in treating primary tumors of the central nervous system have not been reported, preliminary studies with Ade in the D425 Med cells indicated cytotoxicity with a fifty percent effective concentration (EC50) of around 1.0 μM [19]. Based on the available information concerning Ade’s effects in tumor cells, and its physico-chemical properties, the compound seemed an ideal choice for examining our drug delivery approach.

Adenanthin had little to no permeability through the BBB when administered alone; however, co-administration of Ade with HAV6 peptide significantly enhanced its accumulation in the brain. The concentration of Ade in the brain following HAV6 administration was similar for all doses of Ade examined. This could be due to the saturation of the influx of Ade into the brain following the disruption with HAV6 peptide. Of importance for the present study is the observation that concurrent administration of Ade (10 mg/kg dose) and HAV6 peptide resulted in brain concentrations of Ade (>10 μM) that would be expected to be in the therapeutic range required for anti-tumor activity. In addition, it should be noted that despite the large increases in Ade accumulation in the brain, there were no overt clinical signs of toxicity (i.e., no weight loss and no changes in locomotive activity in the mice receiving HAV6 peptide). Furthermore, examination of GFAP and Iba1 expression in the brain showed no enhancement in these neuroinflammatory markers as a result of HAV6 either alone or in combination with Ade. This is an important finding, as increased GFAP and Iba1 expression, markers of reactive astrocytosis and activated microglia, respectively, have been reported with both osmotic and focused ultrasound BBB modulation [43,44].

We had three cohorts of mice (placebo, Ade and Ade + HAV6 peptide) to evaluate the in vivo effect of concurrent administration of Ade and HAV6 peptide in an orthotopic Group-3 MBL model. The HAV6 peptide was not included as a cohort, as the peptide had no effect on Group-3 MBL cells (Figure 4C). The average survival of animals receiving both HAV6 and Ade was 30 days post tumor implantation, compared to 20 and 19 days for the Ade and placebo groups respectively. Furthermore, a complete 5-cycle treatment was only possible in the Ade + HAV6 treatment group. Histological studies also confirm the enhanced response of tumor mice to Ade in the presence of HAV6. Together these findings suggest that the improved delivery of Ade to the brain using HAV6 cadherin peptide to transiently open the BBB impacted favorably on treatment outcome.

In summary, we have shown that HAV6 peptide reversibly modifies BBB permeability allowing for effective delivery of Ade for the treatment of brain tumors. The transient modulation of BBB permeability produced by HAV6 cadherin peptide appeared to be well tolerated. While additional studies are required to validate both the effectiveness of adenanthin and our drug delivery approach in treating Group 3 MBL, the current studies suggest that HAV cadherin peptide can be used to enhance the brain delivery and effectiveness of poorly BBB permeable therapeutic agents. The effects of cadherin peptide based BBB modulation in terms of both the duration and magnitude of BBB opening, coupled with its effectiveness in repeated use settings could prove advantageous for treatment of brain tumors.
